# 120. An open-label comparative trial of SUBA-itraconazole (SUBA) versus conventional itraconazole (c-itra) for treatment of proven and probable endemic mycoses (MSG-15): a pharmacokinetic (PK) and adverse Event (AE) analysis

**DOI:** 10.1093/ofid/ofab466.120

**Published:** 2021-12-04

**Authors:** Peter G Pappas, Andrej Spec, Marisa Miceli, Gerald McGwin, Rachel McMullen, George R R Thompson III

**Affiliations:** 1 University of Alabama at Birmingham, Birmingham, Alabama; 2 Division of Infectious Diseases Washington University in St. Louis, ST LOUIS, MO; 3 University of Michigan, Ann Arbor, Michigan; 4 UC-Davis, Sacramento, CA

## Abstract

**Background:**

C-itra is the drug of choice for treatment of most non-CNS, non-life-threatening forms of endemic mycoses (EM), including histoplasmosis, blastomycosis, coccidioidomycosis, sporotrichosis and talaromycosis. SUBA represents a new formulation of itraconazole that utilizes nanotechnology to improve bioavailability when administered orally. SUBA is formulated as nanoparticles allowing for absorption in the small bowel while not relying on gastric acidity for optimal absorption. MSG-15 is an open-label, comparative clinical trial comparing SUBA to c-itra for the treatment of EM. Herein we report the final PK and AE profiles of these two compounds.

**Methods:**

Subjects with proven and probable EM were eligible this open-label comparative study. The protocol allowed up to 14 d of prior therapy with any antifungal for this episode of EM. Subjects were randomized to receive either SUBA 130 mg po bid or c-itra 200 mg po bid for up to 6 months. Follow up occurred at 7, 14, 28, 42, 84 and 180 d post-enrollment. PK samples were obtained at 7, 14, and 42 d. Clinical assessment, including symptom assessment, AEs, overall drug tolerance, and quality of life were assessed at each visit. We used descriptive statistics for this analysis.

**Results:**

89 subjects with EM entered the trial, including 43 on SUBA and 46 on c-itra. We measured PK serum levels of itra and hydroxyl-itra at days 7, 14, and 42 and these data are depicted in Figures 1-3. There were no significant differences in these levels, including combined itra/hydroxyl-itra levels, among the two study arms. AUC for itra and hydroxyl-itra were similar for both arms. AEs as assessed at each study evaluation were also quite similar among the two study arms. Overall, any AE occurred in 74% vs 85% of SUBA and c-itra recipients, respectively (NS). Drug-related AEs occurred in 35% vs 41% of SUBA and itra recipients, respectively (NS). Most common drug-related AEs included cardiovascular (edema and hypertension), nausea and loss of appetite.

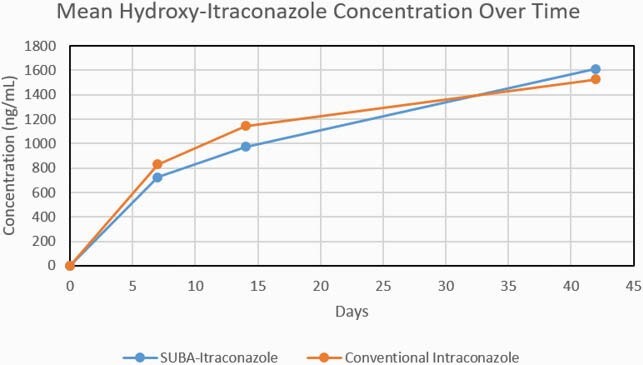

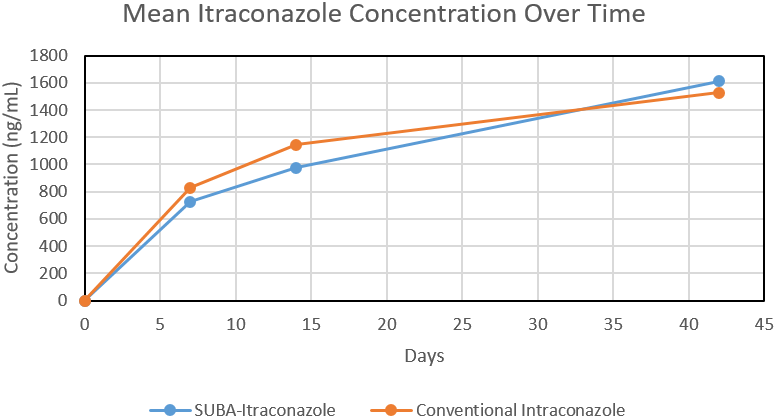

Combined Itraconazole and Hydroxy-itraconazole Concentration Over Time

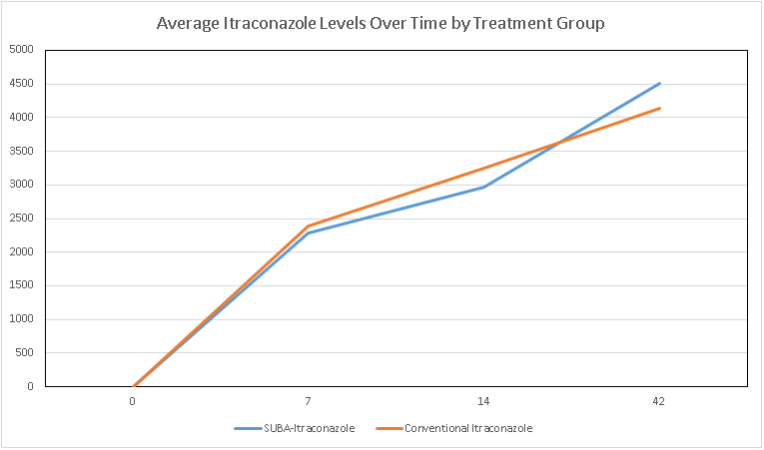

**Conclusion:**

Compared to c-itra, SUBA demonstrates almost identical serum levels despite being dosed at roughly 60% standard dosing for c-itra (130 mg po bid vs 200 mg po bid). SUBA is slightly better tolerated than c-itra, although the specific AEs are similar.

**Disclosures:**

**Peter G. Pappas, MD**, **Astellas** (Research Grant or Support)**Cidara** (Research Grant or Support)**F2G** (Consultant)**Matinas** (Consultant, Scientific Research Study Investigator)**Mayne Pharma** (Research Grant or Support)**Scynexis** (Research Grant or Support) **Andrej Spec, MD, MSCI**, **Mayne Pharma** (Grant/Research Support) **Marisa Miceli, MD**, **SCYNEXIS, Inc.** (Advisor or Review Panel member) **George R. R. Thompson III, III, MD**, **Amplyx** (Consultant, Grant/Research Support)**Appili** (Consultant)**Astellas** (Consultant, Grant/Research Support)**Avir** (Grant/Research Support)**Cidara** (Consultant, Grant/Research Support)**F2G** (Consultant, Grant/Research Support)**Mayne** (Consultant, Grant/Research Support)**Merck** (Scientific Research Study Investigator)**Pfizer** (Advisor or Review Panel member)

